# The Blind Spot of Pharmacology: A Scoping Review of Drug Metabolism in Prematurely Born Children

**DOI:** 10.3389/fphar.2022.828010

**Published:** 2022-02-15

**Authors:** Mette Louise Mørk, Jón Trærup Andersen, Ulrik Lausten-Thomsen, Christina Gade

**Affiliations:** ^1^ Department of Clinical Pharmacology, Copenhagen University Hospital Bispebjerg and Frederiksberg, Copenhagen, Denmark; ^2^ Department of Neonatology, Copenhagen University Hospital Rigshospitalet, Copenhagen, Denmark

**Keywords:** infant, premature, pharmacokinetics, cytochrome P-450 enzyme system, pharmaceutical preparations

## Abstract

The limit for possible survival after extremely preterm birth has steadily improved and consequently, more premature neonates with increasingly lower gestational age at birth now require care. This specialized care often include intensive pharmacological treatment, yet there is currently insufficient knowledge of gestational age dependent differences in drug metabolism. This potentially puts the preterm neonates at risk of receiving sub-optimal drug doses with a subsequent increased risk of adverse or insufficient drug effects, and often pediatricians are forced to prescribe medication as off-label or even off-science. In this review, we present some of the particularities of drug disposition and metabolism in preterm neonates. We highlight the challenges in pharmacometrics studies on hepatic drug metabolism in preterm and particularly extremely (less than 28 weeks of gestation) preterm neonates by conducting a scoping review of published literature. We find that >40% of included studies failed to report a clear distinction between term and preterm children in the presentation of results making direct interpretation for preterm neonates difficult. We present summarized findings of pharmacokinetic studies done on the major CYP sub-systems, but formal meta analyses were not possible due the overall heterogeneous approaches to measuring the phase I and II pathways metabolism in preterm neonates, often with use of opportunistic sampling. We find this to be a testament to the practical and ethical challenges in measuring pharmacokinetic activity in preterm neonates. The future calls for optimized designs in pharmacometrics studies, including PK/PD modeling-methods and other sample reducing techniques. Future studies should also preferably be a collaboration between neonatologists and clinical pharmacologists.

## 1 Introduction

Prematurely born children represent a very fragile subset of neonates, as they often present a complex and challenging pathophysiological condition associated with increased risk of long-term morbidity and mortality. Treatment includes various pharmaceutical agents, yet there is currently insufficient knowledge of gestational age dependent differences in drug metabolism. This potentially puts the preterm at risk of receiving suboptimal drug doses with a subsequent increased risk of adverse or insufficient drug effects (Kearns et al., 2003; O’Hara et al., 2015; van den Anker and Allegaert, 2021).

Although the need for clinical research to identify optimal drug dosing in term and preterm neonates has long been acknowledged as indispensable (Tayman et al., 2011; O’Hara et al., 2015; van den Anker and Allegaert, 2021), the progress has been slow. Recently, data on premature neonates have begun to emerge, and particularly pharmacometric modeling-approaches have added available information on this pediatric subgroup (Smits et al., 2019). However, data is still scarce, and pediatricians are often forced to prescribe medication as off-label or even off-science (Barr et al., 2002; Al-Turkait et al., 2020; Schrier et al., 2020).

## 2 Prematurity

The average human gestation is 40 weeks, and prematurity is defined as being born before 37 weeks of gestation (Engle et al., 2004). The global prematurity rates are approximately 10% but vary from 4 to 5% in some European countries to 15–18% in some parts of Africa and Asia (Blencowe et al., 2012; Chawanpaiboon et al., 2019). It is a complex and challenging pathophysiological condition associated with increased risk of long-term morbidity and mortality (Saigal and Doyle, 2008) and it is the leading cause of death in children under 5 years of age (Harrison and Goldenberg, 2016; Chawanpaiboon et al., 2019). Prematurity can be considered a continuum of organ immaturity with huge differences in presentation, morbidity, and need for treatment at either end of the spectrum and is often sub-categorized in moderate to late preterm (32–36 weeks of gestation), very preterm (28–32 weeks of gestation), and extremely preterm (before 28 weeks of gestation) (Engle, 2004). Neonates can equally be classified per birth weight as low birth, (LBW, <2500 g), very low birth weight (VLBW, <1500 g) and extremely low birth weight (ELBW, <1000 g) (World Health Organization, 2004, 10), but although LBW often is caused by prematurity, it cannot be used directly as a marker of the degree of prematurity as LBW can be caused by intrauterine growth restriction (IUGR).

Although there are wide variations in prematurity survival rates across regions and countries (Helenius et al., 2017), overall survival rates for premature and particularly extremely premature neonates have hugely improved since the 1980s (Glass et al., 2015). Importantly, the limit for early human viability, defined as the earliest gestational age an infant can potentially survive being born at, has steadily dropped and some babies delivered at 24, 23, and even 22 weeks of gestation are now able to survive. A recent study from Sweden reports a 20% survival rate in children born at 22 weeks (Norman et al., 2019) and a Japanese study has found a survival rate of 36% in children born at 22 weeks (Ishii et al., 2013). Case reports of children surviving being born at 21 weeks (Sung et al., 2018; Tiniest Babies Registry) or with birth weight below 250 g have emerged, but survival remains low (Brumbaugh et al., 2019). Whereas the reasons for this progress in prematurity survival certainly are multifactorial, the improvement is believed to be linked to overall improvement in neonatal intensive care, such as development of increasingly better artificial airways and breathing circuits and rational application of mechanical ventilation and airway distending pressure (Glass et al., 2015; Pierrat et al., 2021).

Another major driving factor for the improvement in premature survival rates has been pharmacological advancements, both prenatal, immediately post-natal and post-natal. Antenatally, the administering of a course of corticosteroids to women prior to anticipated preterm birth has been demonstrated to have a marked positive effect of subsequent preterm mortality and morbidity ever since the first randomized controlled trial of betamethasone for the prevention of respiratory distress syndrome in preterm neonates in 1972 (Liggins and Howie, 1972). Subsequently, many clinical trials have demonstrated the effect of antenatal corticosteroids before preterm birth, as summarized in a recent systematic Cochrane review demonstrating its effect on reducing neonatal mortality (RR 0.78, 95% CI 0.70–0.87), respiratory distress syndrome (RR 0.71, 95% CI 0.65–0.78), and cerebral intraventricular hemorrhage (RR 0.58, 95% CI 0.45–0.75) (McGoldrick et al., 2020). Antenatal corticosteroid is a pharmacological cornerstone of prophylactic treatment in preterm birth.

A huge leap in pharmacological treatment of preterm children, and possibly one of the single greatest breakthroughs in treatment of premature children, was the development of exogenous surfactant administration techniques in the 1980s and early 1990s. Since the first successful attempts in 1980 (Fujiwara et al., 1980) using the first artificial preparations, this therapy has become the definitive standard treatment for neonatal respiratory distress syndrome and is believed to be one of the most effective medicines in the health system (Hentschel et al., 2020). As respiratory distress syndrome is the single most important cause of illness and death in preterm children (Stevens et al., 2007; Sardesai et al., 2017), surfactant administration has led to a marked increase in survival and helped lower the limit for early human viability (Rojas-Reyes et al., 2012; Sweet et al., 2019; Hentschel et al., 2020).

Pharmacological treatment remains a cornerstone in postnatal care in preterm children, as demonstrated by a recent review of drug utilization studies in neonatal units that found a high mean number of drugs per infant, with eight studies reporting a very high burden (>30 drugs per infant). Drug use patterns were found to be generally uniform with antibiotics being the most frequently prescribed drug in the neonatal department (Al-Turkait et al., 2020). Naturally, trends in general drug prescription have changed over the years but off-label drug use in premature children is still very common: of the top 50 medications of extremely low birth weight premature infants, only 40% were FDA-labeled for use in infants (Stark et al., 2021). This represents a relative lack of proper pharmacokinetic (PK) studies in premature children and remedying this will likely be a long haul due to the difficulties of conducting clinical PK studies in preterm children.

## 3 Drug Disposition in Premature Neonates

Accurate dosing is essential for a safe and effective pharmacological treatment of premature neonates. An in-depth knowledge of the anatomical and physiological particularities of preterm neonates is therefore crucial for the understanding of the drug pharmacokinetics in this population. Herein, the differences in ontogeny between the extremely, the very and the late preterm neonate should be taken into consideration (Barker et al., 2018; van den Anker et al., 2018).

Also, most aspects of drug disposition are subject to change in case of co-morbidities, which unfortunately relatively often occurs in premature neonates. Sepsis, surgery, and (for moderate and late preterm neonates where such treatment is possible) advanced treatment such as whole body hypothermia (Smith et al., 2019), extracorporeal membrane oxygenation (ECMO), or hemo-dialysis may all significantly alter drug distribution and metabolism.

**FIGURE 1:**
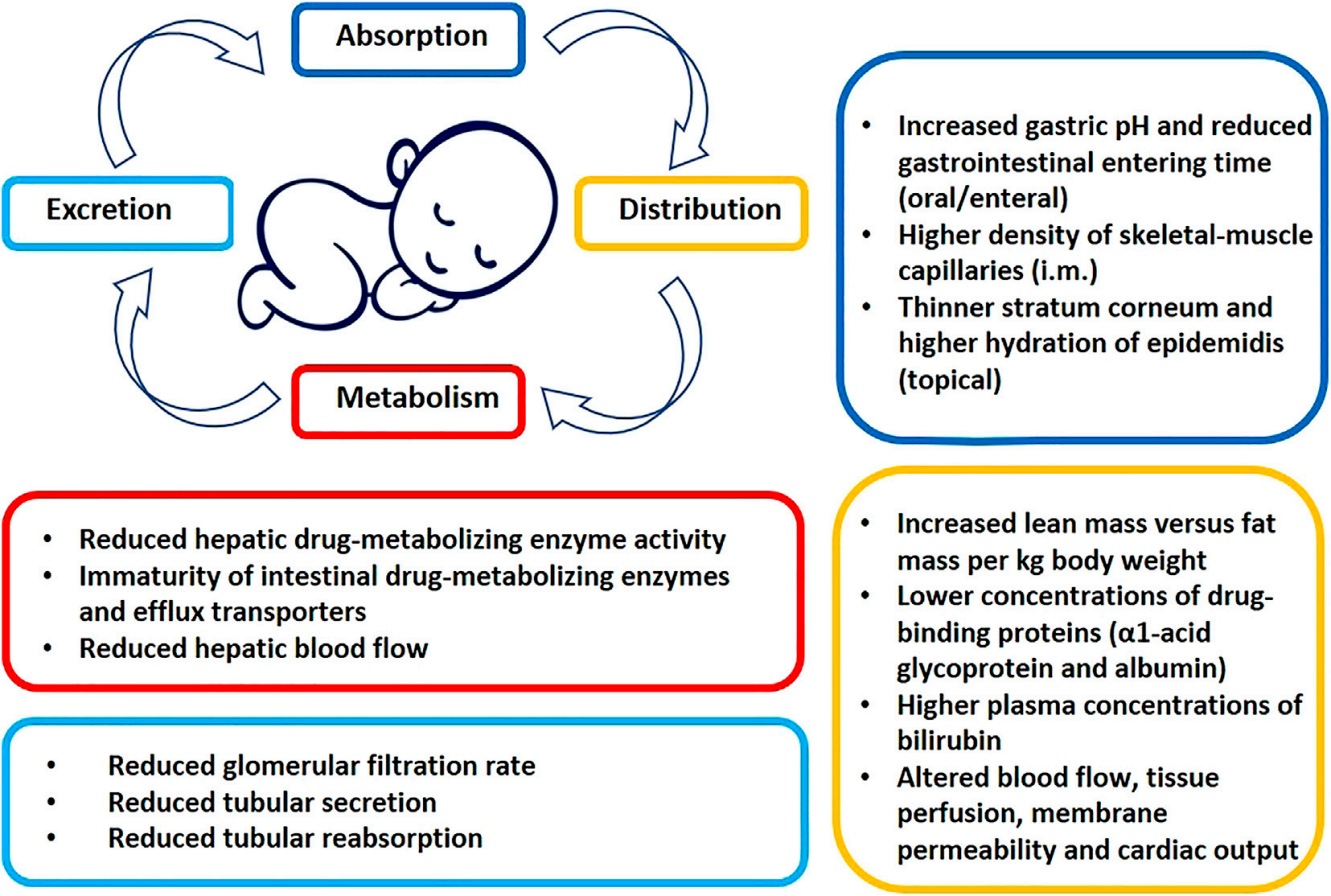
Prematurity associated pato-physiological conditions potentially altering the pharmacokinetics of drugs (see text for details).

A summary of the most important prematurity associated characteristics of pharmacokinetics in preterm neonates is presented in Figure 1 and below.

### 3.1 Absorption

In preterm neonates, the immaturity of absorptive surfaces may influence drug exposure (O’Hara et al., 2015). At present, there is no clear consensus describing the ontogeny of gastric pH and its impact on drug absorption, and gastric emptying time has not been found age dependent (Bonner et al., 2015). However, gastrointestinal abnormalities and dysfunctions in preterm neonates can affect transit time and enteral absorption (Johnson, 2011; van den Anker and Allegaert, 2021). Developmental differences in the activity of intestinal drug-metabolizing enzymes and efflux transporters will most likely affect the exposure of several drugs in preterm neonates, but this area is still not well understood (Kearns et al., 2003; van den Anker and Allegaert, 2021).

The exposure of drugs after rectal administration is generally increased in preterm neonates, e.g., paracetamol, most likely due to developmental immaturity of the hepatic metabolism rather than increased mucosal translocation (Kafetzis et al., 1979; Ruggiero et al., 2014). Due to the relatively higher density of skeletal-muscle capillaries in neonates, water-soluble drugs show an increased intramuscular absorption, e.g., absorption of amikacin (Kafetzis et al., 1979). Whether this is also the case for preterm neonates is unknown, but the example of intramuscular administration of vitamin E acetate in a lipophilic preparation, showed that the ester was never systematically detectable in premature neonates as opposed to E-vitamin delivered in an aqueous preparation (Italian Collaborative Group on Preterm Delivery, 1991).

The presence of a thinner stratum corneum in preterm neonates, a higher body surface area-to-weight ratio as well as higher cutaneous perfusion and hydration of the epidermis places preterm neonates at risk of adverse effects from topical exposures due to an increased absorption e.g. corticosteroids (Borzyskowski et al., 1976; Munro, 1976) and chlorhexidine (Cowen et al., 1979). Application of antiseptic solutions containing alcohols has led to severe burns in premature infants (Brayer et al., 2004). Pulmonary, sublingual, and buccal absorption are not well studied in preterm or term neonates (O’Hara et al., 2015; van den Anker and Allegaert, 2021).

### 3.2 Distribution

In the extremely preterm neonate, total body fat content can be as low as 1% of the total body weight and total body water is decreasing from 85% in preterm neonates to 75% in term neonates (Tetelbaum et al., 2005; Ford and Calvert, 2008) (Sharma et al., 2008). Due to the lower percentage of fat and muscle mass in preterm neonates, drugs that are normally rapidly distributed into the muscle tissue, like fentanyl, will remain in the plasma compartment for a longer time (O’Hara et al., 2015). Significant changes in intra/extracellular body fluid distribution per concurrent weight occurs postnatally during the first week of life. Thus, both gestational age (GA) at birth and postnatal age (PNA) influences total body water content and distribution, and this should ideally be taken into consideration when optimizing individual drug doses (van den Anker and Allegaert, 2021).

At the time of birth, neonates have lower concentrations of the drug-binding proteins α1-acid glycoprotein and albumin when compared to older children (Ehrnebo et al., 1971; Windorfer et al., 1974; Ku and Smith, 2015). The amount of free drug available for distribution will therefore be increased for highly protein bound drugs, e.g., theophylline (Aranda et al., 1976). Effects and toxicity may therefore be obtained at lower total plasma concentrations. Also elevated plasma levels of bilirubin can increase the concentration of unbound drugs by displacing highly bound drugs from protein-binding sites, e.g., ampicillin, benzodiazepine and phenytoin (Fredholm et al., 1975; Tayman et al., 2011; Zwart et al., 2021).

Higher CNS drug concentrations may occur in preterm neonates due to reduced outward drug transport, however, this area still needs to be elaborated (Painter et al., 1981; Liu et al., 2008; Ku and Smith, 2015). Changes in the volume of distribution are also related to changes in blood flow, tissue perfusion, membrane permeability and cardiac output (Kearns et al., 2003; O’Hara et al., 2015; van den Anker and Allegaert, 2021). Furthermore, it should be noticed that pathological circulatory conditions, e.g., a hemodynamically significant persistent ductus arteriosus can also alter the volume of distribution of drugs in preterm neonates (Ku and Smith, 2015; O’Hara et al., 2015).

### 3.3 Metabolism

The major site of drug metabolism is the liver and the drug metabolizing enzymes are broadly divided into phase I and phase II enzymes. The phase I enzymes are responsible for primary oxidation, reduction and hydrolysis processes. The most important group of enzymes involved in phase I metabolism are cytochrome (CYP) P450 enzymes with a major contribution of cytochrome P450 3A4 (Hines and McCarver, 2002). However, while CYP3A4 constitutes 30–40% of the total liver CYP content in adult, CYP3A7 is found to be the major form in human embryonic, fetal and newborn liver (Gow et al., 2001; Hines and McCarver, 2002). In the period from late fetal to early neonatal life, there appears to be a peak in CYP3A7 activity, then a transition in expression and catalytic activity from predominant CYP3A7 to CYP3A4 (de Wildt et al., 1999; Gow et al., 2001).

Phase II enzymes are responsible for conjugate drug molecules to allow excretion. Phase II drug metabolizing enzymes are mostly transferases and include: UDP-glucuronosyltransferases (UGTs), sulfotransferases (SULTs), N-acetyltransferases (NATs), glutathione S-transferases (GSTs) and various methyltransferases (Xu et al., 2005; O’Hara et al., 2015).

A lack of activity of metabolizing enzymes can be responsible for extreme toxicity syndromes (O’Hara et al., 2015). Severe toxicity syndromes in premature infants have been described due to reduced capacity of their metabolic systems, e.g., grey baby syndrome, which is caused by diminished ability to conjugate chloramphenicol and to excrete the active form in the urine (Craft et al., 1974). In addition, gasping syndrome with benzyl alcohol, where benzoic acid cannot be conjugated but is accumulated, causing metabolic acidosis in premature neonates (Gershanik et al., 1982).

Similarly to many other physiological and metabolic processes in newborns, pharmacokinetics also exhibit a relative immaturity that changes postnatally, and for the CYP450 system, it is believed that some CYP450 enzymes are active *in utero* while others do not active until after birth (Gow et al., 2001; O’Hara et al., 2015). When corrected for weight the content of CYP enzymes in fetal livers is 30–60% of adult values and full CYP activity is usually achieved by 2 years of age. Yet, the many physiological and metabolic processes depend not only on postnatal age, but also on gestational age, i.e., degree of maturation, at the time of birth (Gow et al., 2001; Kearns et al., 2003; O’Hara et al., 2015).

Knowledge of maturation of drug metabolizing enzymes is therefore an important factor in determining drug selection in neonates. This is further complicated as various elements of the drug metabolism pathways do not mature at the same rate postnatally (Hines and McCarver, 2002). For example, use of codeine is not appropriate during the first month of life as conversion to morphine *via* CYP2D6 is low resulting in very limited effectiveness (O’Hara et al., 2015). Midazolam is metabolized by CYP3A4 at a slower rate causing increased duration of sedation and early exposure to opioid infusion in the first 3 days was associated with higher risk of adverse outcomes in extremely preterm infants (de Wildt et al., 2010; Shah et al., 2011; Ng et al., 2012; O’Hara et al., 2015). Contrarily, CYP1A2 is induced rapidly after birth with post-natal age rather than post-menstrual age correlating with changes in half-life and clearance (Hines and McCarver, 2002; Schmidt et al., 2006). This rapid induction fits clinically with the lack of toxicity to caffeine seen in even the most premature infants started on caffeine for the prevention or treatment of apnea of prematurity (Schmidt et al., 2006). However, the pattern and timing of post-natal CYP1A2 induction remain unclear.

Maturation rates are difficult to generalize, and enzyme-specific information needs to be determined for an accurate estimate of drug metabolism including clinical conditions such as sepsis and complex surgery, nutritional state and diet (infant formula versus breast milk), polymorphism and even antenatal exposure to cigarette smoke (Czekaj et al., 2005; Blake et al., 2006; Allegaert, 2017; Li et al., 2021).

### 3.4 Excretion

Water soluble drugs with low molecular weight are primarily eliminated by renal excretion. The glomerular filtration rate (GFR) is highly dependent on gestational age and is ranging from 0.6–0.8 to 2–4 ml per minute per 1.73 m^2^ in preterm neonates and term neonates, respectively (Hayton, 2000; Ali et al., 2012). For example, gentamycin is therefore necessitating dosing intervals of 36–48 h in preterm newborns but reduced to 24 h in term newborns (Ali et al., 2012; Fuchs et al., 2014). Also, tubular secretion and reabsorption is reduced in preterm neonates. Tubular secretion has importance for the renal elimination of, e.g., penicillins, cephalosporins, and digoxin (Kearns et al., 2003).

## 4 Challenges in Pharmacokinetic Studies in Premature Neonates

### 4.1 Blind Spots

As the anatomical and physiological characteristics of preterm neonates differ significantly from older children and adults, the process of neonatal pharmacological development becomes very complex. Furthermore, neonatology represents a small sub-field of pharmacology, and as a small target population, neonates are often overlooked.

Pharmaceutical companies largely refrain from proactively investing in the pediatric sector due to both economic and practical considerations, as reported by the EMA (European Medicines Agency and its Paediatric Committee, 2016; Van Driest and Choi, 2019). Even academia-driven research in the population of preterm neonates is limited and often focused on various other areas of clinical research than pharmacology.

### 4.2 Ethical Considerations

The ethical principles of pediatric research are well known (Roth-Cline et al., 2011), but the challenges are even more pronounced in the preterm population. Overall, inclusion of newborns in research should comprise a minimal risk and/or have a potential for direct benefits for the trial participant to be considered ethically acceptable (Barker et al., 2018).

Due to the stressful situation of becoming a parent to a premature child, the required informed parental consent for neonates participating in research, can be difficult to obtain. The process may require repeated discussions with the families, and thus complicate recruitment for studies during the first hours/days of life (O’Hara et al., 2020).

Failure of recruitment of a sufficient number of premature neonates within the planned study period may force investigators to facilitate costly prolongations of the study or even to a premature study termination without reaching the target sample size. Failed drug trials and the general lack of pediatric clinical trials contribute to the high prevalence of off-label use in neonates.

### 4.3 Practical Challenges

Fortunately, relatively few children are born very or extremely preterm, but the scarcity of premature neonates makes it challenging to include this population in PK studies. Traditional PK study designs involve multiple blood samples at fixed intervals and generally require the same number of samples from all subjects taken at the same time (O’Hara et al., 2020). This approach presents practical difficulties in preterm neonates, as repeated blood sampling quickly exceeds the regulated (EMA, 2018; Barker et al., 2018) maximum of 1% at any one time, or 3% within 1 month, amounting to 400 µl or 1.2 ml respectively for a neonate weighing 500 g. Also, the sampling procedure may prove challenging, as even something as mundane as drawing blood often requires experience in the extremely preterm children. Likewise, urine sampling may prove difficult to collect in a standardized manner (Van Driest and Choi, 2019).

## 5 Exploring the Available Data on Premature Neonates: An Example Using Hepatic Drug Metabolism

### 5.1 Methods

A PubMed search was performed on 3 January 2022 for the terms phase I and phase II metabolism in premature children using the following search string: {“Infant, Premature” (mesh) OR “Infant, Extremely Premature” (mesh) OR “Infant, Low Birth Weight” (mesh) OR “Infant, Very Low Birth Weight” (mesh) OR “Infant, Premature, Diseases” (mesh) OR [(preterm OR prematurity OR premature OR prematurely) AND (infant* OR infancy OR baby OR babies OR neonat* OR newborn*)] OR preemi* OR premi OR preterm OR “very low birth weight” OR VLBW OR “low birth weight” OR LBW OR “very low birthweight” OR “low birthweight”} AND {[“Cytochrome P-450 Enzyme System" (mesh) OR “Cytochromes” OR Cytochrome* OR “P450”] OR [“Methyltransferases” (mesh) OR “Sulfotransferases” (mesh) OR “Acetyltransferases” (mesh) OR “Acyltransferases” (mesh) OR “Glucuronosyltransferase” (mesh) OR glucuronidation* OR Methyltransferase* OR Sulfotransferase* OR Acetyltransferase*)]} AND {[humans (Filter)] AND [newborn (Filter)]}.

Manuscripts were reviewed for relevance to the topic of this review, as well as for citations related to the topic of the review by two independent reviewers (MLM and CG). In case of disagreement, a third author (ULT) would arbitrate. This review should however be considered a scoping rather than a systematic review.

### 5.2 Findings

Our search resulted in 1206 hits, of which a total of 70 manuscripts were found relevant to the scoping review (Aranda et al., 1976; Loughnan et al., 1977; Windorfer and Pringsheim, 1977; Pitlick et al., 1978; Onishi et al., 1979; Grygiel and Birkett, 1980; Brazier et al., 1981; Kawade and Onishi, 1981; Mulhall et al., 1983; Tserng et al., 1983; Ribon et al., 1984; Grasela and Donn, 1985; Choonara et al., 1989; RJ et al., 1990; Fujii et al., 1993; Hartley et al., 1993, 1994; Reiter and Stiles, 1993; Brammer and Coates, 1994; Vauzelle-Kervroedan et al., 1996; Sato et al., 1997; Treluyer et al., 1997; Wenzl et al., 1998; Lee et al., 1999; Touw et al., 2000; de Wildt et al., 2001, 2002, 2010; Lowry et al., 2001; Allegaert et al., 2008a, 2008b, 2015; Wade et al., 2008, 2009, 2009; Knibbe et al., 2009; Mugabo et al., 2011; George et al., 2012; Ince et al., 2013; Kim et al., 2013; Le Doare et al., 2013; Wang et al., 2013; Bekker et al., 2014; de Waal et al., 2014; Krekels et al., 2015; Mahmood, 2015, 2017; Ogawa et al., 2015; Pain et al., 2015; Auriti et al., 2016; Cook et al., 2016; Momper et al., 2016; Flint et al., 2017; Hwang et al., 2017; Sohn et al., 2017; Völler et al., 2017; JM et al., 2018, 2018; Anderson and Holford, 2018; Brussee et al., 2018; Leroux et al., 2018; Michelet et al., 2018; Neyro et al., 2018; Tsakiri et al., 2018; Gerhart et al., 2019; Gonzalez et al., 2019; Smith et al., 2019; van Groen et al., 2019).

Overall, the large heterogeneity of the studies with regards to both scope, methodology, and particularly the detail of reporting results, precludes any formal comparison, let alone meta-analyses. In particular, many studies examined neonates, but failed to distinguish between term and preterm neonates. This was the case in more than 40% of the studies. The full spectrum of prematurity was found to be explored from 22 gestational weeks and onwards. Extreme premature neonates (born before 28 GA weeks) were included in less than 55% of these studies.

Furthermore, the age of the children at the time of inclusion and/or sampling (postnatal age, PNA) was unclear in approximately 30% of the studies. In the remaining articles the PNA varied from a few hours to 1 year. The overall mean age at the time of inclusion varied from 4 days to 1.5 months. Keeping in mind that prematurity is defined as being born before 37 weeks of gestation, this logically reduces the actual number of included neonates, who were extremely, very or even late preterm at the time of sampling. These findings elucidate how most PK studies in the youngest pediatric populations are not sufficiently transparent in the presentation of data contributions.

Generally, the gestational- and postnatal ages are displayed in intervals, making an accurate estimation of the degree of prematurity impossible. Additionally, the interpretation of the strength of the contributing data becomes very difficult, as, e.g., a 2 weeks old child that was born at 26 weeks gestational age and a newborn child born at 28 weeks gestational age are not necessarily equal in terms of metabolic maturation.

Most studies (approximately 70%) focused on the phase I metabolism, and the most studied CYP subclasses were CYP3A4, CYP1A2, CYP2E1, CYP2C19, and CYP2C9 either as individual or contributing enzymes. The phase II metabolism systems were studied in approximately 35% of the articles identified [with glucuronidation (notably UDP-Glucuronosyltransferase-2B7)] and sulfation being the most studied.

#### 5.2.1 Phase I Metabolism in Preterm Neonates

In Tables 1– 3 we summarize the Cytochrome P450 subclasses, we found to be the most studied *in vivo* in preterm neonates, i.e., 3A4, 1A2, and 2C9/2C19.

#### 5.2.2 CYP3A4

Identified studies of CYP3A4 activity are displayed in Table 1. Midazolam was predominantly reported as an *in vivo* probe for CYP3A4 activity, and the clearance values reported illustrated a clear tendency towards reduced CYP3A4 activity in preterm neonates as compared to full born neonates and older children (Burtin et al., 1994; Vauzelle-Kervroedan et al., 1996; Lee et al., 1999; de Wildt et al., 2001, 2002; Ogawa et al., 2015; Brussee et al., 2018; JM et al., 2018; van Groen et al., 2019). The largest study identified was a population-based study (Burtin et al., 1994) included plasma samples from 197 neonates (gestational age 26–42 weeks, postnatal age 0–10 days) on artificial ventilation. Here clearance was found to be directly proportional to birth weight and gestational age, but not postnatal age (between 0 and 10 days).

**TABLE 1:** Summary of studies exploring CYP3A (4/5) activity in preterm neonates.

References	Premature (N)	GA, range	PNA, range	BW, range	Substrate	Dose	Clearance parameter	Clearance	Clearance premature
Burtin et al. (1994)	Min. 96*	26–42	0–10	700–5200	Midazolam	0.032–1.6 mg/kg (IV bolus)	Total CL	Mean 1.2 (SD ± 0.96) ml/kg/min	↓
Vauzelle-Kervroedan et al. (1996)	7	31.4–36.5	1–14	1540–2700	Cortisol	- (endogenous)	6 βOHF/FF ratio	Mean 7.2 (SD ± 1.5)	(↑)
Lee et al. (1999)	60	24–31	2–15	523–1470	Midazolam	0.1 mg/kg (IV bolus)	Total CL	Mean 1.0 (SD ± 0.2) ml/kg/min	↓
De Wildt et al. (2001)	24	26–34	3–11	760–1630	Midazolam	0.1 mg/kg (IV bolus)	Total CL and 1-OH-M/M (AUC_0−t_) ratio	Mean 2.3 (SD ± 1.5) ml/kg/min and 0.09 (<0.001–1)	↓
De Wildt et al. (2002)	15	26–31	3–13	Mean 1076 (SD ± 240)	Midazolam	0.1 mg/kg (PO or IV bolus)	CL/F and 1-OH-M/M (AUC_0−t_) ratio	2.7 (range 0.7–15.5) ml/kg/min and 0.03 (<0.01–0.96)	↓
Brussee et al. (2018)	Min. 55*	ND	1–44	770–3700	Midazolam	0.1 mg/kg (IV 30 min infusion)	Total CL	**	**
Ogawa et al. (2015)	34	24–32.9	1–37	598–1868	Doxapram	0.2 mg/kg (IV)	Total CL	0.698 L/kg/h	↓
Brussee et al. (2018)	37	26–34	3–11	770–2030^1^	Midazolam	0.1 mg/kg (PO or IV)	Hepatic CL	1.62 L/h	↓
Groen et al., 2019	Unclear*	23.9–41.4	4.2–343.7	2600–8900^1^	Midazolam (^14^C-marked)	111 Bq/kg; 37.6 ng/kg (IV)	Total CL	Median 1.8 (range 0.7–6.7) ml/kg/min	↓

1 Weight at sample time.

*Exact number of premature children included has not been specified.

**The model over-estimated clearance and was not found applicable to predict midazolam CL in critical ill preterm neonates.

BW, birth weight in grams; Bq, Becquerel; CL, clearance; GA, gestational age at birth in weeks; IV, intravenous; N, number of included premature neonates; ND, not defined; PNA, postnatal age at start of sampling in days; PO, orally.

#### 5.2.3 CYP 2C9 and - 2C19

Identified studies of CYP2C19 and CYP2C9 activity are displayed in Table 2. Only phenobarbital was found reported as an *in vivo* probe for CYP2C9 (Pitlick et al., 1978; Ribon et al., 1984; Grasela and Donn, 1985; MP et al., 1989; Touw et al., 2000; Völler et al., 2017), although additional minor metabolism occurs *via* CYP2C19 and CYP2E1 (approximately 5%). A general trend towards reduced phenobarbital clearance was observed in premature neonates. In a recent population study, a PK model was developed based on data sharing from former studies, and the maturation of clearance was predicted to be dependent on both body weight and postnatal age in preterm neonates.

**TABLE 2:** Summary of studies exploring CYP2C9 (rows 1–6) and CYP2C19 (rows 7–8) activity in preterm neonates.

References	Premature (N)	GA, range	PNA, range	BW, range	Substrate	Dose	Clearance parameter	Clearance	Clearance premature
Pitlick et al. (1978)	Unclear*	30–40	<2	1350–2850	Phenobarbital	LD, 20 mg/kg (IV)	Total CL (T_1/2_)	-	↓
						MD, 5 mg/kg/day			
Ribon et al. (1984)	17	28–37	<1	1250–3000	Phenobarbital	5 mg/kg (IM) or 10 mg/kg (IV)	Total CL (T_1/2_)	-	↓
De Carolis et al., 1989	Unclear*	27–37	ND	800–3090	Phenobarbital	LD 20 mg/kg (IV)	Total CL (T_1/2_)	-	↓
						MD 5 mg/kg/15 h			
Grasela and Donn, (1985)	Min 46*	24–42	1–16	600–3620	Phenobarbital	LD, 20 mg/kg	Total CL	Mean 0.0047 (±19%) L/h/kg	↔
						MD, 5 mg/kg			
Touw et al. (2000)	Unclear*	26 + 6–41 + 4	ND	590–4070	Phenobarbital	LD 23 ± 11 mg/kg	Total CL/total CL per kg body weight	Mean 9.3 (SD ± 4.9) ml/h/mean 4.3 (SD ± 1.1)	↑
						MD 51 mg/kg/day			
Völler et al. (2017)	Min. 25*	24–42	0–22	450–4400	Phenobarbital	LD 20 mg/kg	Total CL	Mean 0.0091 (±9%) L/h	↓
						MD 3.9 mg/kg			
Loughnan et al. (1977)	4	32–36	2–18	760–2950	Phenytoin	12 mg/kg (IV)	Total CL (T_1/2_)	-	↓
Ward et al. (2010)	37	23–41	9.1–137.2	2018–4550	Pantoprazol	0.6 or 1.2 mg/kg/day (PO)	CL/F	Mean 0.21 (SD ± 0.12) L/h/kg (1.25 mg)	↓
								Mean 0.23 (SD ± 0.21) L/h/kg (2.5 mg)	

*Exact number of premature children included has not been specified.

BW, birth weight in grams; CL, clearance; GA, gestational age at birth in weeks; IM, intramuscular; IV, intravenous; LD, loading dose; MD, maintenance dose; N, number of included premature neonates; ND, not defined; PNA, postnatal age at start of sampling in days; PO, orally.

The activity of CYP2C19 was investigated by use of the substrate’s phenytoin and pantoprazole (Loughnan et al., 1977; Ward et al., 2010). Latest was investigated by Ward et al., in 2010, who found oral clearance of pantoprazole reduced in preterm neonates. Oral clearance increased with increased postnatal age, but no apparent trend was seen for postmenstrual age.

#### 5.2.4 CYP1A2

Primarily theophylline was used as substrate in all the identified *in vivo* studies investigating CYP1A2 activity in preterm neonates (Grygiel and Birkett, 1980; Brazier et al., 1981; Tserng et al., 1983; Lowry et al., 2001; Kim et al., 2013; Sohn et al., 2017; Jiang et al., 2021), which are displayed in Table 3. CYP1A2 was generally found associated to postnatal age rather than postmenstrual age and to birth weight. Furthermore, well-known genetic polymorphism-associated differences in CYP1A2 activity were not yet found expressed in the preterm population.

**TABLE 3:** Summary of studies exploring CYP1A2 activity in preterm neonates.

References	Premature (N)	GA, range	PNA, range	BW, range	Substrate	Dose	Clearance parameter	Clearance	Clearance premature
Grygiel and Birkett. (1980)	6	28–32	ND	800–1620	Theophylline	4.5 (±0.04) mg/day (PO)	Theophylline urin metabolite ratio	-	↓
Brazier et al. (1981)	2	32	1–9	1360, 1380	Theophylline	3 mg/kg/8 h (PO)	Total CL and Theophylline urin metabolite ratio	-	↓
Tserng et al. (1983)	9	26–32	4–39	780–2050	Theophylline	LD 6.6 mg/kg (IV)	Theophylline urin metabolite ratio	-	↓
						MD 2.6 mg/kg/8 h			
Lowry et al. (2001)	3	24, 28, 31	56, 21, 0	880, 1060 and 1800	Theophylline	2 mg/kg/12 h and 2.5 mg/kg/12 h (all overdoses)	Total CL	0.01, 0.02 and 0.05 L/h/kg	↓
Kim et al. (2013)	100	24.3–35.7	2.8–79.1	500–2900^1^	Theophylline	Dose not specified	Total CL	0.16 (SD ± 20%) L/h	↓
Sohn et al. (2017)	104	24 + 2–35 + 5	5–74	540–2500	Amiphylline Theophylline	LD 8 mg/kg (IV/PO)	Total CL, Theophylline urin metabolite ratio	0.5 (SD ± 0.29) ml/min/kg	↓
				540–2900^1^		MD 1,3–3 mg/kg/8 or 12th h		0.34 (SD ± 0.28) ml/min/kg	
Jiang et al. (2021)	17	26–32	4–43	750–2400	Caffeine	5.01 ± 0.56 mg/kg	Total CL	7.3 (SD ± 2.5) ml/h	↓

1 Weight at sample time.

BW, birth weight in grams; CL, clearance; GA, gestational age at birth in weeks; IV, intravenous; LD, loading dose; MD, maintenance dose; N, number of included premature neonates; ND, not defined; PNA, postnatal age at start of sampling in days; PO, orally.

#### 5.2.5 CYP2E1

Three studies investigating the *in vivo* CYP2E1 activity were identified. Isoniazid (Bekker et al., 2014) and paracetamol (Cook et al., 2016; Flint et al., 2017) were the substrates studied. A markedly reduced isoniazid clearance was noted in neonates with low GA and LBW. None of the studies using paracetamols as substrate succeeded in defining the CYP2E1 maturity, probably because the contribution of the CYP2E1 pathway is minimal, albeit important due to formation of the toxic metabolite NAPQI, for the metabolism of paracetamol.

#### 5.2.6 CYP2D6

*In vivo* CYP2D6 metabolism has been investigated in premature neonates using tramadol as substrate (Allegaert et al., 2008a; 2008b). Here, PMA and CYP2D6 polymorphisms (Li et al., 2021) was found to determine the O-demethylation activity in the preterm neonates.

#### 5.2.7 Phase II Metabolism in Premature Neonates

Twenty studies investigating the *in vivo* phase II metabolism were identified (Windorfer and Pringsheim, 1977; Mulhall et al., 1983a; Choonara et al., 1989; Hartley et al., 1993, 1994; H et al., 1993; Reiter and Stiles, 1993; Brammer and Coates, 1994; Sato et al., 1997; Wenzl et al., 1998; Wade et al., 2008, 2009; Knibbe et al., 2009; Krekels et al., 2015; Mahmood, 2015; Auriti et al., 2016; Cook et al., 2016; Flint et al., 2017; MF et al., 2017; Leroux et al., 2018; Gerhart et al., 2019). A large heterogenitet was found and several substrates were used and included chloramphenicol, morphine, fluconazole, lorazepam, micafungin, paracetamol, and mefenamic acid. Overall, the activity of the phase II metabolis, pathways were found reduced in preterm neonates. Herein, morphine metabolism by UGT2B7 was found closely related to body weight as opposed and post-natal age (day 1–10) (Knibbe et al., 2009), and fluconazol clearance by UGTB7was found to increase with increase with BGA, PNA and PMA (Wade et al., 2008).

### 5.3 Interpretation

The understanding of developmental pharmacology in infants and children has increased significantly since the seminal review by Kearns et al. (2003) and new data have been added in both neonates in general (Allegaert, 2017) and in premature neonates (van den Anker and Allegaert, 2021). Furthermore, pharmacometrics modelling approaches are now, despite limited data, being used to support neonatal and pediatric drug development as well as commonly used off-label drugs. The insight into ontogeny of, e.g., the phase I metabolism of extreme premature neonates has previously been based on, e.g., fetal samples (Kearns et al., 2003). However, with a limit of early human viability, that is, constantly improving, PK data on the extreme premature neonates remain unreasonable scarce.

The results from the present study illustrate the difficulties in obtaining data from the very youngest and smallest neonates. Particularly, it is challenging to explore include patients shortly after birth and consequently the early stages of xenobiotic biotransformation ontogeny remain relatively underexplored.

This is exemplified by our findings, as most studies lacked detailed information, e.g., on the number of very and extremely preterm neonates. Importantly, the post-natal age at sampling for the included patients were often not specified, making definitive interpretation of these early stages of xenobiotic biotransformation very difficult. Finally, details on the number of samples deriving from the preterm neonates was also seldom reported.

As expected, we did find an overall clear tendency of an immature, and thus reduced, drug metabolism in the preterm infants when compared to term neonates and older children. This was the case for virtually all reported enzyme-systems. However, as we also found a large heterogeneity in the studies, including methodological differences in the studies pathways and the use of both non-compartmental- and population PK methods, we cannot perform any direct comparison or formal meta-analysis of the reported findings.

## 6 Possible Solutions and Future Directions

Neonatology has been fortunate to experience some major leaps in pharmacology treatments for premature children but game-changing drugs like surfactant are rare. However, new therapeutic products are increasingly being studied for neonatal diseases and advances are constantly being made. These current and future advances must integrate the increased knowledge of the ontogeny of organ and enzyme systems in premature children. Thereby we can construct optimized models that takes into consideration the normal maturation of neonates (van den Anker and Allegaert, 2021).

As illustrated, there is still a lack of data from extremely premature children sampled shortly after birth, a testament to the practical and ethical challenges. Neonatal pharmacological research could benefit from increased representation of multidisciplinary neonatal clinicians on relevant committees to streamlining of ethics and governance procedures for multi-site studies. This will likely require dedicated time and support as clinician time for additional service outside of clinical care is often unfunded and burdensome. Similarly, the practicalities of inclusion of extremely premature children immediately after birth may be tackled by increased use of antenatal parental consent (Memon et al., 2020).

Obviously, the development of sparse plasma/blood sampling and analyzing techniques improve the feasibility of including the very small preterm neonates. This fact, and the use of left-over or opportunistic samples from routine sampling will yield a higher inclusion rate. Pharmacometric modeling approaches have reduced the high number of samples needed, but by combining these modern techniques with a higher inclusion rate, a high level of detailing will be possible By use of already existing modeling methods and optimal sampling design, parameter estimation with maximal precision is possible (Smits et al., 2021). However, refinement of these methods will be important for the future.

From a regulatory point of view, it would be highly desirable if continued improvements on targeted and efficient clinical trial designs in neonates became standard in pediatric drug development. Herein, PK and PD characterization in the pediatric subgroup of neonatology should be considered from the earliest drug development stages.

Furthermore, development of microdosing studies present an attractive alternative to overcome ethical and analytical challenges in phenotyping studies. Microdoses are subtherapeutic doses (typically < 100th of therapeutic dose) that are unlikely to elicit any pharmaceutical response or side effects. The microdosing approach has been validated for, e.g., midazolam in preterm neonates (van Groen et al., 2019).

The use of international multicenter studies, pediatric trial networks, diverse databases, biomarkers, and integration of Real-world Data, use of artificial intelligence and machine learning such as text mining and deep-learning models to extract relevant information from electronic patient records is likely to further advance neonatal pharmacology (Mulugeta et al., 2018; Goulooze et al., 2020; Smits et al.). However, models of any kind should be developed wisely, bearing in mind that the risk of poor performance is particularly high if certain age groups such as preterm neonates are underrepresented or absent in the data set used to develop the model (Mulugeta et al., 2018; Goulooze et al., 2020; Smits et al.).

## 7 Conclusion

This review highlights the insufficiencies in the current knowledge of the maturation of drug metabolizing enzymes in preterm neonates, and particularly in the very or extremely preterm neonates. We illustrate the overall heterogeneous approach to measuring and presenting the phase I and II pathways metabolism in preterm neonates, often with use of exclusively opportunistic sampling—a testament to the practical and ethical challenges in measuring pharmacokinetic activity in preterm neonates.

With the advances in overall neonatal intensive care, the limit for possible survival in extremely preterm neonates has improved. Consequently, neonates with increasingly lower gestational age at birth now require care, including intensive pharmacological treatment. This calls for optimized designs of future pharmacometrics studies, preferably as multi-site/international collaboration between neonatologists and clinical pharmacologists that will allow for integration of all available techniques, including low volume plasma/blood analysis techniques, pharmacokinetic modeling, “Big Data”, and even machine learning.

The understanding of the impact of growth, development, and organ maturation on the absorption, distribution, metabolism, and excretion of drugs in neonates, infants, children, and adolescents has progressed tremendously in recent decades, but it has mainly been driven by a few, but dedicated researchers; it is now time for the broader pharmacological scientific community to turn its gaze toward the most premature children.
